# Immediate effects of extracorporeal radial pressure wave therapy on dystonia, static plantar pressure distribution, and balance in patients with Parkinson’s disease

**DOI:** 10.3389/fnagi.2025.1539225

**Published:** 2025-05-19

**Authors:** Zhiqin Xu, Xin Huang, Qingqing Liu, Bo Peng, Lingxiang Zhou, Xinghua Dong, Guangyan Dai, Xiaoxia Zhu, Zepeng Weng, Di Lei, Xi Chen

**Affiliations:** ^1^Department of Rehabilitation Medicine, The First Affiliated Hospital, Sun Yat-sen University, Guangzhou, China; ^2^Department of Rehabilitation Medicine, Zhongshan Torch Development Zone People's Hospital, Zhongshan, China

**Keywords:** extracorporeal radial pressure wave therapy, Parkinson’s disease, plantar pressure, dystonia, balance

## Abstract

**Background:**

Extracorporeal radial pressure wave therapy (ERPWT) has emerged as a potential non-invasive treatment for various musculoskeletal and neurological disorders. This study investigates the immediate effects of ERPWT on dystonia, static plantar pressure distribution, and balance in patients with Parkinson’s disease (PD).

**Methods:**

Thirteen participants with PD were recruited from the Department of Rehabilitation Medicine at the First Affiliated Hospital of Sun Yat-sen University. After obtaining informed consent, clinical information was recorded, and measurements of lower limb muscle tone, stiffness, and elasticity, as well as static plantar pressure distribution and center of pressure (COP), were measured. Participants subsequently received a single session of ERPWT administered to the bilateral plantar fascia. Following ERPWT, participants were re-evaluated immediately after treatment.

**Results:**

After ERPWT, significant decreases were observed in muscle tone, stiffness, and elasticity of the achilles tendon on the more PD-affected side and the anterior aspect of the planta on both feet (*p* < 0.05). Additionally, significant reductions in COP measures were noted post-treatment (*p* < 0.001). However, no significant changes were found in muscle tone, stiffness, and elasticity of the achilles tendon on the less PD-affected side or in the anterior tibialis and gastrocnemius. Furthermore, there were no significant changes in static plantar pressure distribution (*p* > 0.05).

**Conclusion:**

These findings indicate that a single session of ERPWT applied to the bilateral planta fascia can yield immediate beneficial effects in reducing dystonia symptoms and improving balance in patients with Parkinson’s disease. This therapy may serve as an adjunctive treatment to address motor symptoms in this population. Further research is warranted to explore the long-term effects of ERPWT and its mechanisms of action in PD patients.

## Introduction

Parkinson’s disease (PD) is one of the most prevalent neurodegenerative disorders worldwide, primarily characterized by motor symptoms that are often accompanied by various non-motor symptoms, significantly impacting the quality of life. The motor manifestations of PD typically include bradykinesia, gait abnormalities, postural dysregulation, tremors, and muscle rigidity (Hayes, 2019; Roldán-Kalil et al., 2025). As the disease progresses, patients may experience forward flexion of the body, abnormal plantar pressure distribution, and decreased balance function, which can lead to falls and increased healthcare burdens. Pathologically, PD is marked by the loss of dopamine-producing neurons in the substantia nigra, resulting in reduced dopaminergic input to the striatum and the consequent overactivation of output from the basal ganglia (Magrinelli et al., 2016; Redgrave et al., 2010). Dysfunction of the basal ganglia and the resulting abnormalities in neural circuitry are thought to be key contributors to the motor symptoms of PD (Nguyen et al., 2024).

PD patients exhibit abnormal plantar pressure distribution and impaired postural control, which lead to atypical gait patterns and the development of festinating gait (Jeon et al., 2008). Abnormal distribution of plantar pressure destabilizes the patients’ center of gravity, compelling them to adjust their bilateral foot pressure to maintain balance and prevent falls (Bassani et al., 2014). Furthermore, elevated muscle tone affects the skeletal muscles throughout the body. For instance, increased muscle tone in the upper limbs can result in the “dystonic hand,” while increased tone in the lower limbs may lead to the “dystonic foot” (Ashour and Jankovic, 2006). These conditions can adversely affect limb movement and overall quality of life.

Due to impaired automatic postural responses and anticipatory postural adjustments, the ability of PD patients to regulate their balance autonomously is significantly diminished. Elevated muscle tone may also represent a compensatory mechanism, such as increased axial muscle tone serving as a strategy to prevent falls in these individuals. Balance deficits often manifest early in the course of PD (Maetzler et al., 2012; Mancini et al., 2011), and their underlying mechanisms are complex and influenced by multiple factors, potentially involving neural pathways beyond dopaminergic systems (Chung et al., 2010; Müller et al., 2013). Until now, the effects of drug treatments and surgical interventions for myospasm are not very satisfactory (Guo et al., 2022).

Extracorporeal radial pressure wave therapy (ERPWT) is a widely utilized physical treatment modality characterized by its biomechanical effects mediated through pressure waves. This therapy employs a handheld device to generate radial pressure waves, which propagate in a spherical diffusion pattern to target painful or injured tissues. The process stimulates cellular metabolism, promotes angiogenesis, reduces inflammation, and breaks down calcified deposits. ERPWT evolved from Extracorporeal Shockwave Therapy, a common physical therapy intervention that primarily exerts biological effects through cavitation, thereby enhancing microcirculation and alleviating adhesion in joint tissues (Mariotto et al., 2009). Compared to traditional shockwave generators, ballistic radial wave devices produce a lower peak positive pressure with a significantly longer rise time, yet radial pressure waves remain capable of inducing acoustic cavitation (Cleveland et al., 2007; Császár et al., 2015).

ERPWT is widely utilized in the treatment of musculoskeletal disorders (Mariotto et al., 2009). Current research indicates that ERPWT significantly alleviates pain and functional limitations in patients with plantar fasciitis by improving pain and biomechanical properties (Al-Siyabi et al., 2022). Furthermore, an increasing number of studies suggest that ERPWT shows promising potential for rehabilitation in cases of central and peripheral nervous system injuries (Guo et al., 2022; Cao et al., 2024). ERPWT can effectively reduce muscle spasticity, significantly lowering the degree of upper and lower limb spasticity in stroke patients and enhancing their overall functionality (Moon et al., 2013; Troncati et al., 2013; Yang et al., 2024). Additionally, Yang et al. (2017) found that ERPWT improves the elasticity and stiffness of the wrist extensor tendons in patients with lateral epicondylitis.

Despite these findings, there is currently a lack of research on the effects of ERPWT on abnormal plantar pressure distribution, muscle tone disorders, and balance functions in PD patients. Understanding these effects is crucial for developing effective therapeutic strategies that can enhance patient outcomes. Therefore, this study aims to explore the immediate effects of ERPWT on muscle tone, stiffness, and elasticity, as well as static plantar pressure distribution and balance in PD patients.

## Materials and methods

### Participants

This study employed a self-controlled before-and-after design, enrolling a total of 13 PD patients from the Rehabilitation Department of the First Affiliated Hospital of Sun Yat-sen University. The inclusion and exclusion criteria were as follows. Inclusion Criteria: (1) Diagnosis of primary Parkinson’s disease. (2) Hoehn and Yahr stages 1–3. (3) Stable medical condition. (4) No cognitive impairment. Exclusion Criteria: (1) Presence of lower limb musculoskeletal disorders. (2) Coexisting neurological conditions, such as stroke.

The study was approved by the Institutional Ethical Committee for Clinical Research and Animal Trials of the First Affiliated Hospital of Sun Yat-sen University (approval No. [2024]655) and was conducted in accordance with the Declaration of Helsinki. Written informed consent was obtained from all participants prior to the experiment.

### Procedure

Initially, participants underwent the Unified Parkinson’s Disease Rating Scale part III (UPDRS-III) and Hoehn and Yahr stage assessment to evaluate the severity of their condition. Additionally, balance, static plantar pressure distribution, and muscle tone, stiffness, and elasticity were assessed. After these evaluations, all participants received a single session of ERPWT targeting the bilateral plantar fascia. Post-treatment evaluations of muscle tone, stiffness, and elasticity, static plantar pressure distribution, and balance were then conducted. The above procedures are all performed during the patient’s “ON” state of the disease.

### Muscle tone, stiffness, and elasticity assessment

A digital muscle palpation device (MyotonPRO, Estonia) was used to evaluate the muscle tone, stiffness and elasticity. This study assessed muscle tone, stiffness, and elasticity in PD patients at rest. Participants were positioned either supine or prone, with their feet hanging off the edge of the bed during the prone position, allowing the lower limbs to remain relaxed. The assessments focused on the anterior tibialis, gastrocnemius (both medial and lateral head), achilles tendon, and the anterior aspect of the planta. The Myoton probe was positioned perpendicular to the skin surface, and vertical pressure was applied until the indicator light changed from red to green. The device then applied five short pulses (with a 1 s interval) to induce damped oscillation in the muscle. Specific operational instructions can be found in the device manual.^1^

The following parameters were obtained directly from the MyotonPRO: Frequency (Hz), Stiffness (N/m), and Decrement. Non-neural tone, or state of tension, is recorded as frequency of the damped oscillations. Stiffness reflects the muscle’s ability to resist an external force that modifies its shape. Logarithmic Decrement characterizes elasticity or the dissipation of natural oscillation (Agoriwo et al., 2022).

### Static plantar pressure distribution

The plantar pressure measurements were conducted using the Gaitview plantar pressure measurement system and Gaitview analysis software (alFOOTS AFA-50 system, Seoul, South Korea). During the static plantar pressure assessment, participants were instructed to relax for 5 min before standing barefoot on the designated measurement area with their feet placed side by side. They were asked to keep their arms hanging naturally, gaze forward, and maintain relaxed and steady breathing. Once stable, they stood in a natural posture for approximately 10 s, during which pressure readings from different areas of the foot were recorded. Measurements were taken three times for each participant.

The parameters recorded included average plantar pressure (kPa), forefoot pressure (kPa), rearfoot pressure (kPa), plantar pressure proportion, forefoot proportion, and rearfoot proportion for both the more PD-affected and less PD-affected feet.

### Balance test

This study utilized the Nintendo Wii Balance Board (WBB; Nintendo Co., Kyoto, Japan) to assess participants’ standing center of pressure (COP). The device operates at a sampling frequency of 100 Hz and transmits data to computer acquisition software via Bluetooth. With advantages including portability, strong operability, and reliable data quality, the WBB has been widely adopted for COP assessment and measurement (Cui et al., 2024; Hao et al., 2021). The testing tasks included double-leg stable stance, double-leg unstable stance, single-leg stance on the more PD-affected side, and single-leg stance on the less PD-affected side. To simulate an unstable surface, a soft mat was placed on the balance board. The duration for double-leg stance was set at 20 s, while single-leg stance was set at 15 s, with each task repeated three times. Adequate rest periods were provided to prevent participant fatigue during the tests. One investigator stood beside each participant to ensure safety and prevent falls. For participants with severe balance impairments who were unable to maintain single-leg stance, only the double-leg stance tasks were performed.

The methods for processing COP measures were adapted from previous studies (Cui et al., 2024; Hao et al., 2021). The data analysis was performed using MATLAB software (MathWorks, Natick, MA, United States, 2023a). The center of mass position was calculated based on ground reaction forces and moments, followed by filtering with a 20 Hz low-pass, second-order, zero-lag Butterworth filter. Additionally, the mean value was removed from the filtered data. Subsequently, the center of mass displacement was analyzed using conventional methods in both the anterior–posterior (AP) and mediolateral (ML) directions. COP measures included the sway mean velocity (MV, mm/s) in both the anterior–posterior (AP) and medial-lateral (ML) directions, sway length (SL, mm), and sway area (SA, mm^2^). MV refers to the average velocity of the COP displacement in the AP or ML direction. SL represents the total path length of COP movement in both directions over a given time period. SA is defined as the area of the 85% confidence ellipse fitted to the COP trajectory.

### Extracorporeal radial pressure wave therapy

The ERPWT was administered using a therapy device (HEMA, S2, Zhuhai). The device has a maximum energy flux density of 0.9 mJ/mm^2^, a pulse width of 3.7 μs, and a treatment diameter of 15 mm. Participants were positioned supine with their feet relaxed. The therapy was applied to both the plantar fascia, toe flexor muscles, and the toe extensors on both sides. The treatment parameters for unilateral therapy included an intensity of 2 bar, frequency of 8 Hz, delivering a total of 1,500 impulses. Each individual pulse contained an energy of 159 mJ, resulting in a cumulative energy delivery of 238.5 J. The impulse per single pulse measured 1.31*10^−4^ N·s, with an instantaneous peak power reaching 43 kW per pulse.

### Data analysis

Statistical analyses were conducted using SPSS version 20.0 (IBM SPSS Inc., Chicago, IL, United States). The Shapiro–Wilk test was employed to evaluate the normality of data distribution. A paired sample *t*-test or a non-parametric test was used to assess the differences in data before and after the intervention for the subjects. A significance level of *α* < 0.05 was maintained to determine statistical significance.

## Results

All 13 participants completed the intervention and assessments. Among them, two individuals were unable to maintain the single-leg stance for 15 s, resulting in the exclusion of single-leg stance COP measures for those participants. The demographic and clinical information of the 13 PD participants is presented in Table 1.

**Table 1:** Descriptive characteristics of the participants.

Variable	Participants (*n* = 13)
Sex (male/female)	7/6
Age (years)	62.23 ± 7.60
BMI (kg/m^2^)	22.83 ± 2.63
HY stage	2.23 ± 0.58
UPDRS-III	20.80 ± 8.70
More PD-affected side (right/left)	10/3

BMI, body mass index; HY Stage, Hoehn and Yahr stage; UPDRS-III, Unified Parkinson’s Disease Rating Scale part III; PD, Parkinson’s disease.

### Muscle tone, stiffness, and elasticity

**Figure 1:**
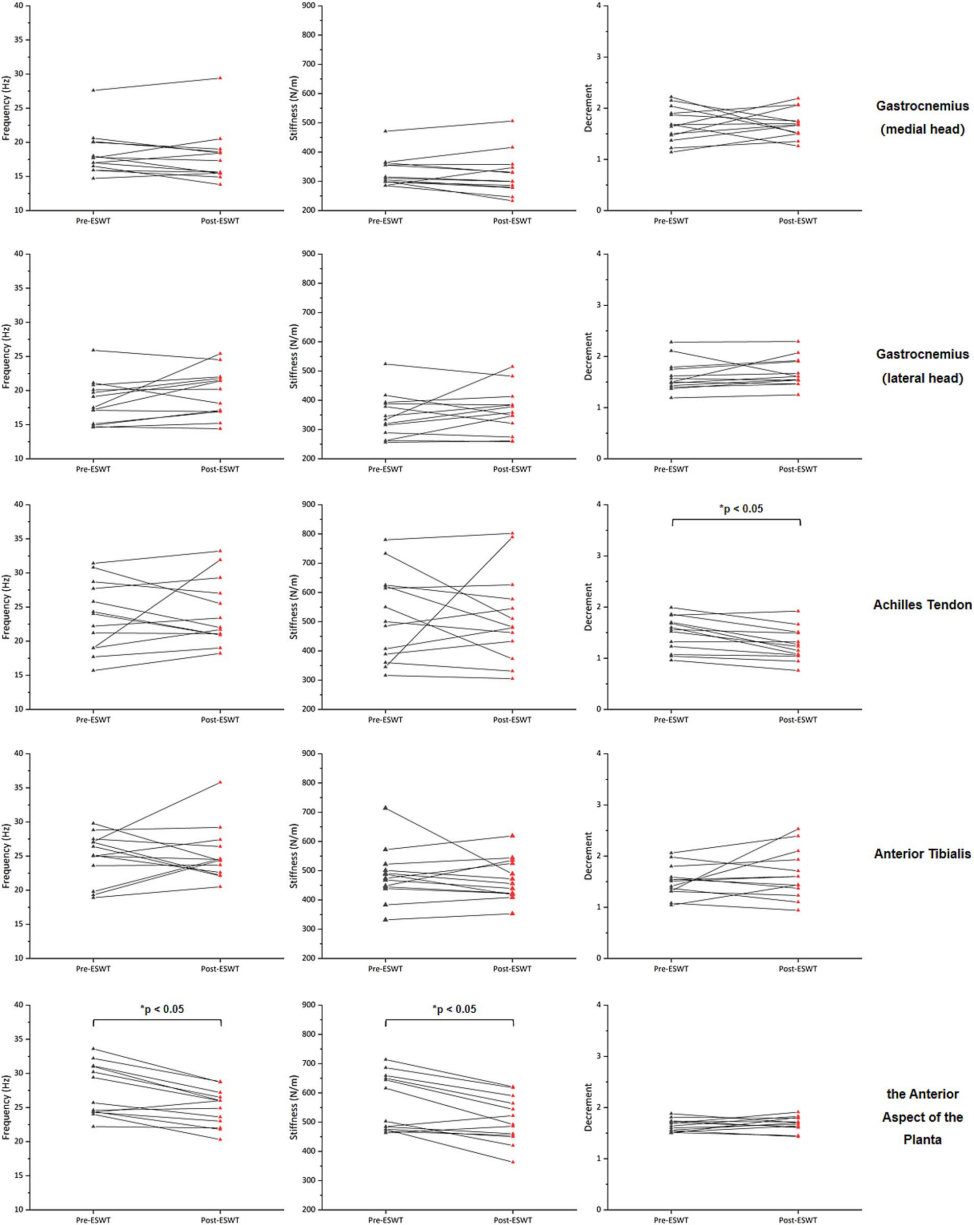
Changes in muscle tone, stiffness, and elasticity on the more PD-affected side before and after ERPWT.

**Figure 2:**
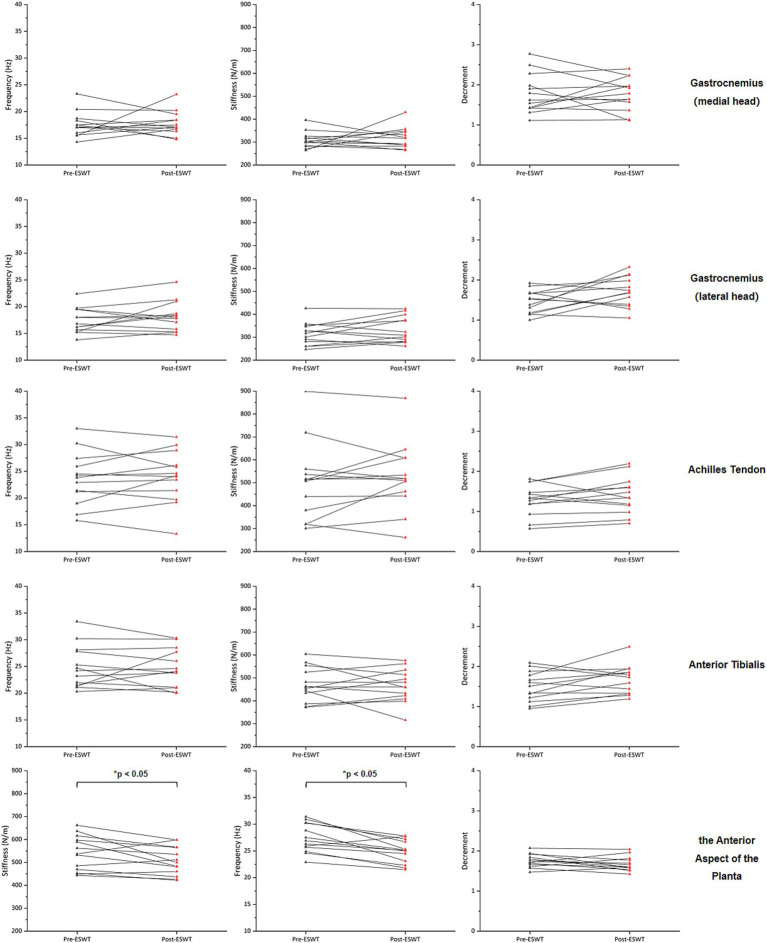
Changes in muscle tone, stiffness, and elasticity on the less PD-affected side before and after ERPWT.

**Figure 3:**
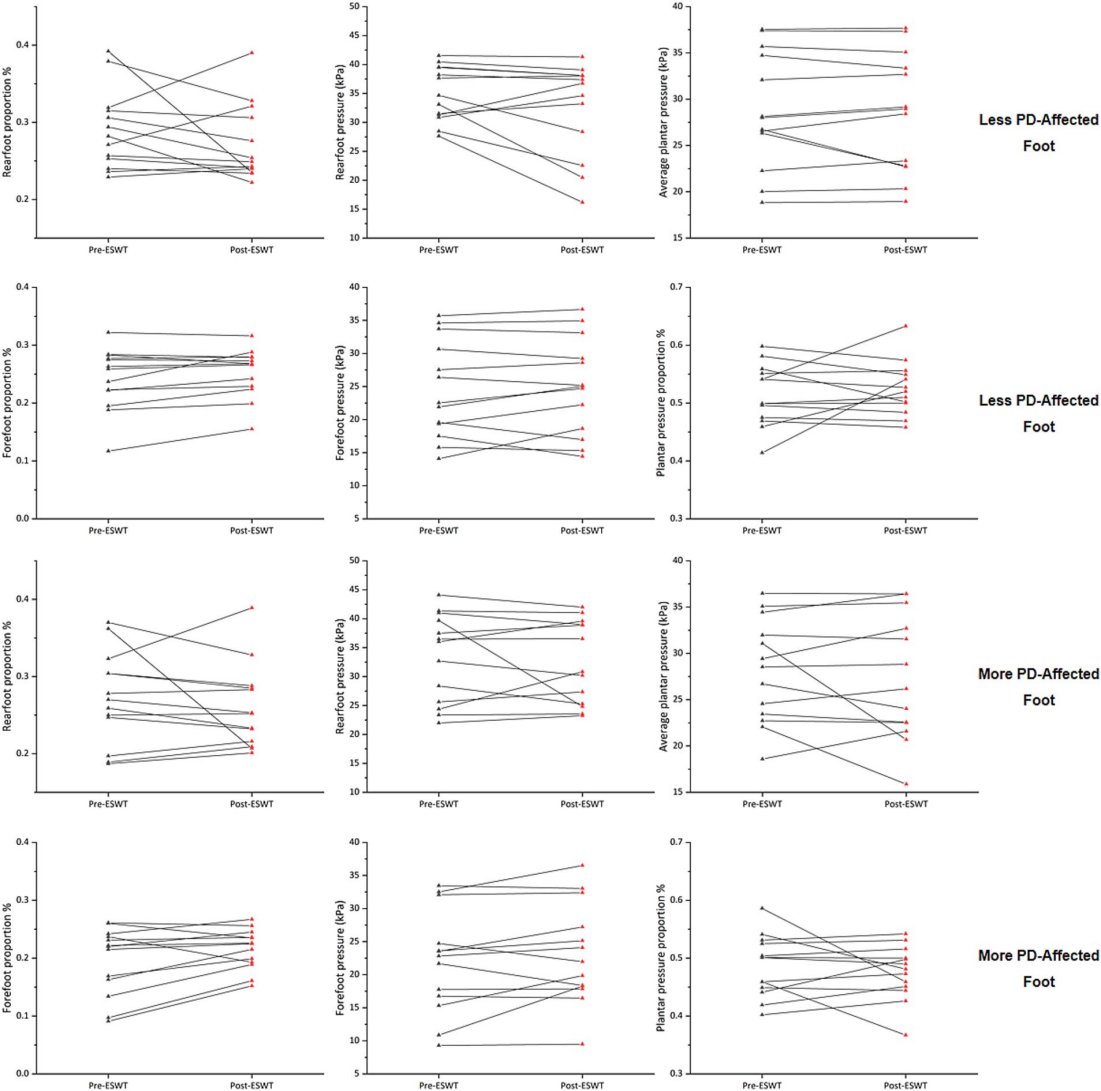
Changes in static plantar pressure distribution before and after ERPWT.

After ERPWT, a significant decrease in the decrement was observed in the achilles tendon on the more PD-affected side (*p* < 0.05) (Figure 1; Supplementary material). Additionally, significant reductions in frequency and stiffness were noted for the anterior aspect of the planta on both feet (*p* < 0.05) (Figures 2, 3). However, no significant changes were found in muscle tone, stiffness, or elasticity of the achilles tendon on the less PD-affected side, as well as in the anterior tibialis and gastrocnemius (*p* > 0.05) (Figures 1, 2; Supplementary material).

### Plantar pressure distribution

Following the ERPWT intervention, there were no significant changes in average plantar pressure, forefoot pressure, rearfoot pressure, plantar pressure proportion, forefoot proportion, or rearfoot proportion on both the two feet when compared to pre-intervention levels (*p* > 0.05) (Figure 3; Supplementary material).

### Balance

After ERPWT, all COP measures showed significant improvement in double-leg stable stance, double-leg unstable stance, and single-leg stance on both the more and less PD-affected legs compared to pre-intervention measurements, with statistical significance (*p* < 0.001), as shown in Table 2.

**Table 2:** Comparison of COP measures under different tasks before and after ERPWT [M (IQR)].

Task	COP measures	Before ERPWT	After ERPWT	*Z*	*p*
		*n* = 13	*n* = 13		
Double-leg stable stance	MV_ML	14.15 (11.71)	11.22 (3.75)	−8.04	<0.001
	MV_AP	22.58 (13.25)	13.79 (7.92)	−6.54	<0.001
	SL	534.09 (371.52)	369.18 (178.80)	−7.34	<0.001
	SA	499.67 (490.34)	441.15 (249.79)	−6.97	<0.001
Double-leg unstable stance	MV_ML	15.90 (17.30)	13.77 (6.68)	−17.95	<0.001
	MV_AP	23.03 (14.32)	15.98 (7.96)	−19.11	<0.001
	SL	553.69 (380.37)	475.32 (220.43)	−19.00	<0.001
	SA	2412.96 (1699.64)	1077.00 (1044.46)	−13.93	<0.001
Single-leg stance on the more PD-affected side	MV_ML	29.67 (18.77)	24.45 (10.07)	−14.33	<0.001
	MV_AP	26.04 (16.00)	21.85 (13.01)	−15.90	<0.001
	SL	665.71 (412.14)	547.22 (229.93)	−15.93	<0.001
	SA	1002.28 (869.22)	544.46 (885.78)	−6.53	<0.001
Single-leg stance on the less PD-affected side	MV_ML	34.97 (10.29)	29.13 (9.00)	−16.07	<0.001
	MV_AP	38.89 (14.06)	27.63 (10.18)	−16.20	<0.001
	SL	899.37 (308.21)	662.60 (141.27)	−16.07	<0.001
	SA	967.24 (1284.26)	984.26 (806.57)	−13.84	<0.001

ERPWT, extracorporeal radial pressure wave therapy; MV, mean velocity; AP, anterior–posterior; ML, medial-lateral; SL, sway length; SA, sway area.

### Adverse effects

Throughout the ERPWT procedure, all subjects experienced mild, tolerable prickling sensations, with no significant adverse reactions reported.

## Discussion

To our knowledge, this is the first study to investigate the application of ERPWT in PD patients, focusing on its effects on muscle tone, stiffness, and elasticity, as well as on static plantar pressure distribution and balance function. ERPWT is a widely recognized rehabilitation treatment for musculoskeletal disorders and is increasingly utilized in the functional rehabilitation of neurological conditions. It has shown promise in improving functional impairments resulting from central and peripheral nervous system injuries (Cao et al., 2024). Our findings indicate that a single session of moderate-intensity ERPWT on bilateral planta can improve muscle tone, stiffness, and elasticity in achilles tendon on the more PD-affected side and the anterior aspect of the planta, as well as enhance static balance stability in PD patients. However, it does not significantly impact static plantar pressure distribution or muscle tone, stiffness, and elasticity in areas distant from the treatment site. Given that correcting abnormal plantar pressure distribution, improving gait, and enhancing balance stability are crucial for the rehabilitation of PD patients, it is vital to identify methods that can rapidly improve lower limb function in these population for effective recovery.

### Effects of ERPWT on muscle tone, stiffness, and elasticity in PD patients

Muscle rigidity is one of the hallmark symptoms of PD, characterized by increased tone in both agonist and antagonist muscles, commonly referred to as “lead-pipe rigidity.” The etiology of dystonia in PD is not fully understood. However, some studies suggest that it is associated with abnormal neuronal firing and oscillatory activity in the basal ganglia. Additionally, the interplay between the cerebellum and basal ganglia contributes to the development of muscle tone disorders in PD (Dijkstra et al., 2014; Vitek, 2002; Weinberger et al., 2012). Multiple neurotransmitter pathways, including dopaminergic and cholinergic systems, are implicated in these processes (Bonsi et al., 2011; Dang et al., 2012).

ERPWT has been shown to effectively reduce hypertonicity in patients with upper motor neuron syndromes without significant side effects. Trompetto et al. (2009) reported that ERPWT improved pain and spasticity scores in patients with secondary dystonia due to basal ganglia lesions, along with a transient improvement in writing function. Additional studies have demonstrated that ERPWT effectively alleviates spasticity and enhances motor function in patients with stroke and cerebral palsy (Troncati et al., 2013; Yang et al., 2024; Gonkova et al., 2013). Clinical research by Agoriwo et al. (2022) indicates that data obtained from MyotonPRO measurements are reliable for assessing the impact of medical and physical interventions on muscle tone in PD patients. In our study, a single session of moderate-intensity ERPWT significantly reduced muscle spasm in the achilles tendon on the more-affected side and the anterior aspect of the planta in PD patients. However, it did not affect the muscle tone, stiffness, or elasticity in the anterior tibialis and gastrocnemius. This finding suggests that the therapeutic effects of a single session of moderate-intensity ERPWT may be limited and primarily localized to the treated muscle groups.

From a peripheral perspective, shock wave therapy exerts a direct therapeutic effect on tendinopathies by influencing the rheological properties of tendon fiber degeneration and chronic hypertonia. Specifically, the mechanical vibrations may modulate muscle spindle sensitivity and alter the viscoelastic characteristics of muscle tissue (Kapoor, 2012; Speed, 2004). Additionally, ERPWT exhibits neuroblocking effects, suppressing fibrosis in chronically spastic muscles and improving muscle viscoelasticity (Kenmoku et al., 2012; Othman and Ragab, 2010; Radwan et al., 2008). From a central nervous system perspective, Leone and Kukulka (1988) observed that mechanical compression on tendons can reduce spinal excitability. Furthermore, vibrational stimulation of tendons via ERPWT has been found to decrease the excitability of spinal motor neurons. ERPWT also enhances neuronal nitric oxide synthase activity, inducing nitric oxide synthesis and modulating neurotransmitter release (Ciampa et al., 2005; Lindgren et al., 2013). The coordinated action of these peripheral and central mechanisms is likely responsible for the observed changes in muscle tone. Despite these promising findings, research on the application of ERPWT for muscle tone disorders in PD patients remains limited, and further investigation into its underlying mechanisms for improving muscle tone in this population is warranted.

### Effects of ERPWT on static plantar pressure distribution in PD patients

Due to impaired muscle tone, PD patients often develop various foot deformities, which reduce the contact area between the foot and the ground, subsequently diminishing their mobility (Ni et al., 2024). Research by Silvia et al. indicates that during static standing, PD patients exhibit a greater distribution of body weight towards the hindfoot, with a significant correlation observed between weight release in the plantar area and COP sway (Silvia Aparecida et al., 2023). During walking, PD patients show reduced heel strike duration and early weight bearing on the forefoot, leading to increased pressure in the forefoot and midfoot, which contributes to a forward shift in their center of gravity and results in a shuffling gait (Kimmeskamp and Hennig, 2001; Nieuwboer et al., 1999; Spaulding et al., 2013).

In our study, a single session of moderate-intensity ERPWT applied to the bilateral planta did not significantly alter the static plantar pressure distribution in PD patients. Previous research indicates that while ERPWT can improve pain scores, reduce skin temperature at the heel, and enhance functional scores in patients with plantar fasciitis, its effects on plantar fascia thickness and pressure distribution are limited (Güzel et al., 2024; Wang et al., 2024). However, findings in the literature are inconsistent, with some studies reporting that ERPWT can modify plantar pressure distribution in patients with plantar fasciitis (Brachman et al., 2020; Hsu et al., 2013). Additionally, the efficacy of treatment is closely related to the total dosage of ERPWT delivered (Gollwitzer et al., 2007), and varying treatment intensities may yield different therapeutic outcomes (Güzel et al., 2024; Wang et al., 2019). In our study, the use of a single session of ERPWT may explain the lack of significant changes in static plantar pressure distribution.

### Effects of ERPWT on balance in PD patients

Posturography is a widely used objective method for measuring balance sway in standing subjects, with increased sway indicating a reduced balance stability (Błaszczyk et al., 2007; Jacobs et al., 2006). Our study demonstrates that ERPWT can enhance stability during both stable and unstable double-leg stances, as well as improve stability during single-leg stances on stable surfaces, thereby improving balance function in PD patients.

Research indicates that approximately 92% of PD patients exhibit balance abnormalities within 15 years of disease onset (Hely et al., 2008). While balance impairment is a hallmark of disease progression to Hoehn and Yahr stage 3, previous studies have shown that abnormal body sway and balance dysfunction can occur early in the disease, increasing the risk of falls (Kim et al., 2013). The regulation of balance involves several anatomical structures, including the cerebral cortex, brainstem, and cerebellum. Abnormalities in dopaminergic and cholinergic pathways contributing to balance disturbances in PD (Müller et al., 2013; Revilla et al., 2013). Additionally, muscle weakness, altered postural reflexes, variable postural responses to perturbations, and diminished anticipatory postural adjustments further exacerbate postural instability and the fall risk in this population (Chagdes et al., 2016).

Plantar pressure is not only related to gait stability but also closely linked to balance functions (Huang et al., 2022). Additionally, the plantar flexor muscles play a crucial role in preventing excessive ankle dorsiflexion and maintaining balance, while also influencing the motion and stability of the knee and hip joints (Skinner et al., 2019). Research by Ni et al. (2024) and Huang et al. (2022) indicates that using botulinum toxin to treat foot dystonia in PD can normalize plantar pressure distribution and enhance lower limb motor function and balance abilities. In our study, muscle tone, stiffness, and elasticity assessment following ERPWT intervention was performed prior to balance evaluation. Although our study found that ERPWT did not significantly alter static plantar pressure distribution in PD patients, it did reduce muscle spasm in the planta and achilles tendon before balance evaluation, which may improve ankle-foot function. This improvement, in turn, could enhance the ability to respond to postural adjustments and ultimately improve balance stability. However, although assessments were performed during the “ON” state in early-to-mid stage PD patients, confounding effects of disease progression and dopaminergic medications on muscle tone and balance outcomes remain possible.

### Limitations

While this study represents the first investigation of the effects of ERPWT on dystonia, plantar pressure distribution, and balance in PD patients, several limitations warrant consideration. First, our intervention involved a single session of ERPWT, which may have influenced the observed outcomes. The absence of cumulative dosing effects restricts our ability to assess the full therapeutic potential of ERPWT. Increasing the number of treatment sessions might yield more significant results. Second, our assessments were limited to static plantar pressure distribution and static balance stability, and did not encompass evaluations of dynamic functional performance, such as gait analysis or dynamic plantar pressure distribution. Third, although our sample size is sufficient for preliminary exploration, the substantial functional variability among PD patients across different Hoehn and Yahr stages suggests that future studies should consider stratifying or categorizing participants to draw more reliable conclusions. Moreover, the underlying mechanisms through which ERPWT affects dystonia, plantar pressure distribution, and balance in PD patients require further investigation. Last but not least, this pilot study has limited evidence. Future research will incorporate control groups, blinding procedures, and standardized experimental designs to provide more robust evidence.

## Conclusion

This study provides a timely contribution to non-pharmacological PD management research. A single session of moderate-intensity ERPWT applied to the bilateral planta can effectively improves muscle tone, stiffness, and elasticity in the achilles tendon of the more PD-affected side and the anterior aspect of the planta, as well as enhances static balance stability in PD patients. However, it does not significantly affect static plantar pressure distribution or muscle tone, stiffness, and elasticity in areas distant from the treatment site. Further research is necessary to elucidate the specific mechanisms by which ERPWT influences functional impairments in PD patients.

The observed acute effects highlight the need for further investigation through randomized controlled trials with extended follow-ups, mechanistic studies incorporating neurophysiological measures (e.g., H-reflex, electromyography), and multisession protocols to assess cumulative benefits. Additionally, gait analysis should be included to evaluate dynamic stability outcomes. Future research should also explore dose–response relationships and long-term functional impacts to strengthen clinical applicability.

## Data Availability

The raw data supporting the conclusions of this article will be made available by the authors, without undue reservation.

## Footnotes

^1^ https://www.myoton.com/UserFiles/Updates/MyotonPRO_User_Manual.pdf
