# Supplementary material for: Immediate effects of extracorporeal radial pressure wave therapy on dystonia, static plantar pressure distribution, and balance in patients with Parkinson’s disease
Source: Front Aging Neurosci. 2025 May 19;17:1539225. doi: 10.3389/fnagi.2025.1539225 (PMC12127289; doi:10.3389/fnagi.2025.1539225)
Supplement: Supplementary file 1 [file Table_1.DOCX]

Supplementary Table 1 Comparison of Muscle Tone, Stiffness, and Elasticity Before and After ERPWT [M(IQR)]

| **Variable** | **Site** | **Side** | **Before ERPWT**  **n = 13** | **After ERPWT**  **n = 13** | **Z** | **P** |
| --- | --- | --- | --- | --- | --- | --- |
| Frequency | Gastrocnemius (medial head) | Less PD-affected | 17.10(2.70) | 17.10(2.50) | -0.105 | 0.916 |
|  |  | More PD-affected | 17.70(3.85) | 17.30(3.35) | -0.979 | 0.328 |
|  | Gastrocnemius (lateral head) | Less PD-affected | 16.80(4.00) | 18.00(4.30) | -1.049 | 0.294 |
|  |  | More PD-affected | 17.50(5.45) | 20.20(5.00) | -1.642 | 0.101 |
|  | Achilles Tendon | Less PD-affected | 23.80(6.55) | 24.20(6.90) | -0.489 | 0.624 |
|  |  | More PD-affected | 24.00(9.20) | 22.00(7.25) | -0.175 | 0.861 |
|  | Anterior Tibialis | Less PD-affected | 24.20(6.50) | 24.10(7.05) | -0.594 | 0.552 |
|  |  | More PD-affected | 25.10(5.60) | 24.40(4.50) | -0.140 | 0.889 |
|  | Anterior Aspect of Planta | Less PD-affected | 26.90(4.95) | 25.10(4.25) | -2.903 | ***0.004*** |
|  |  | More PD-affected | 25.70(6.75) | 26.00(4.35) | -2.762 | ***0.006*** |
| Stiffness | Gastrocnemius (medial head) | Less PD-affected | 302.00(39.50) | 317.00(61.50) | -0.078 | 0.937 |
|  |  | More PD-affected | 311.00(61.00) | 300.00(74.00) | -0.874 | 0.382 |
|  | Gastrocnemius (lateral head) | Less PD-affected | 317.00(79.00) | 309.00(106.5) | -1.153 | 0.249 |
|  |  | More PD-affected | 334.00(115.00) | 358.00(101.00) | -0.804 | 0.422 |
|  | Achilles Tendon | Less PD-affected | 513.00(198.50) | 518.00(156.50) | -0.734 | 0.463 |
|  |  | More PD-affected | 500.00(238.50) | 482.00(198.50) | -0.314 | 0.753 |
|  | Anterior Tibialis | Less PD-affected | 460.00(129.00) | 460.00(108.50) | -0.078 | 0.937 |
|  |  | More PD-affected | 474.00(69.50) | 456.00(110.50) | -0.280 | 0.780 |
|  | Anterior Aspect of Planta | Less PD-affected | 537.00(145.5) | 499.00(117.00) | -2.202 | ***0.028*** |
|  |  | More PD-affected | 503.00(179.00) | 492.00(124.50) | -2.691 | ***0.007*** |
| Decrement | Gastrocnemius (medial head) | Less PD-affected | 1.57(0.53) | 1.79(0.82) | -2.062 | ***0.039*** |
|  |  | More PD-affected | 1.68(0.55) | 1.68(0.41) | -0.245 | 0.807 |
|  | Gastrocnemius (lateral head) | Less PD-affected | 1.52(0.50) | 1.69(0.68) | -1.678 | 0.093 |
|  |  | More PD-affected | 1.50(0.35) | 1.61(0.41) | -1.924 | 0.054 |
|  | Achilles Tendon | Less PD-affected | 1.33(0.55) | 1.34(0.61) | -1.363 | 0.173 |
|  |  | More PD-affected | 1.23(0.45) | 1.56(0.62) | -2.824 | ***0.005*** |
|  | Anterior Tibialis | Less PD-affected | 1.51(0.66) | 1.73(0.57) | -1.818 | 0.069 |
|  |  | More PD-affected | 1.51(0.40) | 1.60(0.70) | -0.664 | 0.507 |
|  | Anterior Aspect of Planta | Less PD-affected | 1.73(0.24) | 1.60(0.23) | -1.399 | 0.162 |
|  |  | More PD-affected | 1.64(0.20) | 1.70(0.18) | -0.699 | 0.485 |

ERPWT: Extracorporeal radial pressure wave therapy.

Supplementary Table 2 Comparison of Static Plantar Pressure Distribution Before and After ERPWT [M(IQR)]

| **Variable** | **Side** | **Before ERPWT**  **n = 13** | **After ERPWT**  **n = 13** | **Z** | **P** |
| --- | --- | --- | --- | --- | --- |
| APP (kPa) | Less PD-affected | 27.99(10.91) | 28.95(11.46) | -0.245 | 0.807 |
|  | More PD-affected | 28.53(10.13) | 26.18(12.03) | -0.175 | 0.861 |
| FP (kPa) | Less PD-affected | 22.53(13.74) | 25.05(13.37) | -0.524 | 0.600 |
|  | More PD-affected | 22.81(12.36) | 21.96(11.75) | -1.433 | 0.152 |
| RP (kPa) | Less PD-affected | 34.67(8.46) | 36.74(12.67) | -1.363 | 0.173 |
|  | More PD-affected | 36.01(15.56) | 30.82(14.24) | -0.314 | 0.753 |
| PPP (%) | Less PD-affected | 0.50(0.13) | 0.52(0.06) | -0.140 | 0.889 |
|  | More PD-affected | 0.50(0.08) | 0.48(0.06) | -0.140 | 0.889 |
| FP (%) | Less PD-affected | 0.26(0.07) | 0.27(0.05) | -1.853 | 0.064 |
|  | More PD-affected | 0.22(0.09) | 0.23(0.05) | -1.960 | 0.050 |
| RP (%) | Less PD-affected | 0.28(0.07) | 0.25(0.08) | -1.223 | 0.221 |
|  | More PD-affected | 0.27(0.09) | 0.25(0.07) | -0.769 | 0.442 |

ERPWT: Extracorporeal radial pressure wave therapy; APP (kPa): average plantar pressure (kPa); FP (kPa): forefoot pressure (kPa); RP (kPa): rearfoot pressure (kPa); PPP (%): plantar pressure proportion; FP (%): forefoot proportion; RP (%): rearfoot proportion.
